# A Bayesian Hierarchical Spatial Model to Correct for Misreporting in Count Data: Application to State-Level COVID-19 Data in the United States

**DOI:** 10.3390/ijerph19063327

**Published:** 2022-03-11

**Authors:** Jinjie Chen, Joon Jin Song, James D. Stamey

**Affiliations:** Department of Statistical Science, Baylor University, Waco, TX 76798-7140, USA; jinjie_chen@alumni.baylor.edu (J.C.); joon_song@baylor.edu (J.J.S.)

**Keywords:** COVID-19, under-reporting, Bayesian, spatial, over-reporting

## Abstract

The COVID-19 pandemic that began at the end of 2019 has caused hundreds of millions of infections and millions of deaths worldwide. COVID-19 posed a threat to human health and profoundly impacted the global economy and people’s lifestyles. The United States is one of the countries severely affected by the disease. Evidence shows that the spread of COVID-19 was significantly underestimated in the early stages, which prevented governments from adopting effective interventions promptly to curb the spread of the disease. This paper adopts a Bayesian hierarchical model to study the under-reporting of COVID-19 at the state level in the United States as of the end of April 2020. The model examines the effects of different covariates on the under-reporting and accurate incidence rates and considers spatial dependency. In addition to under-reporting (false negatives), we also explore the impact of over-reporting (false positives). Adjusting for misclassification requires adding additional parameters that are not directly identified by the observed data. Informative priors are required. We discuss prior elicitation and include R functions that convert expert information into the appropriate prior distribution.

## 1. Introduction

Coronavirus disease 2019 (COVID-19) is an infectious disease caused by Severe Acute Respiratory Syndrome Coronavirus 2 (SARS-CoV-2). The first known case was reported and confirmed in Wuhan, China, in December 2019. Since then, the disease has spread globally, leading to an ongoing pandemic. According to data from Johns Hopkins University, since the initial spread of the disease in the United States in March 2020, there have been over 762,000 deaths and about 47 million contractions as of mid-November, 2021, more than any other country.

Misclassification is a common problem in public health count data. Misclassification leads to both biased parameter estimates for regression coefficients and also to interval estimates that are too narrow when the uncertainty in the model is not fully accounted for. Common approaches for correcting under-reporting include the censored likelihood method [1,2] and a hierarchical count framework [3,4,5,6]. Recently, Stoner et al. [7] extended the model of Winkelmann et al. [3] to correct under-reporting of tuberculosis in Brazil. In their paper, spatial random effects were also incorporated to account for neighborhood dependency. The Bayesian model they consider is highly flexible and could be applied to a variety of public health scenarios. In our paper, we extend the model and apply it to COVID-19 data. We extend the model in two ways. One, because of the complexity of the model, we replace the Besag-York-Mollié (BYM) model [8] with BYM2 [9,10] and use the Stan programming language of Gelman et al. [11]. We found our implementation with Stan sped up the computation considerably compared to the results using the Nimble package developed by de Valpine et al. [12]. Secondly, since there were likely some false positives (over-reporting) in the COVID-19 data, we extend the model to allow for a background rate of false positives. By including both under-reporting and false positives, the model we propose is overparameterized. Thus, the prior elicitation procedure becomes particularly important, and model robustness to prior sensitivity should be checked. We include R functions to assist with prior elicitation and consider different prior combinations in our example to show robustness to moderate changes in the prior parameters. This can be viewed as a sensitivity analysis for the model to see if inferences are impacted by varying degrees of false positives.

In this paper, we adopt a retrospective methodology to explore the impact of under-reporting (false negatives) and over-reporting (false positives) on statistical models of state-level COVID-19 cases in the early stages of the pandemic in the United States. There have been many studies supporting the argument that the COVID-19 cases were considerably under-estimated early in the pandemic. For example, in a community seroprevalence study in Los Angeles County, the prevalence of antibodies for SARS-CoV-2 was 4.65% (bootstrap 95%, confidence interval 2.52–7.07%), indicating that approximately 367,000 (198,890–557,998) of adults had SARS-CoV-2 antibodies, which is substantially greater than the 8430 cumulative number of confirmed infections in the county as of 10 April 2020 [13]. In other words, the case reporting rate was only 2.30% (1.51–4.24%). Another study of SARS-CoV-2 antibodies by Bendavid et al. [14] implies that by early April, the case reporting rate was approximately 1.85% (1.10–4.00%) in Santa Clara County, CA. Furthermore, Hortacsu et al. [15] estimate 4–14% of actual infections had been reported in the US up to 16 March 2020. Ribeiro et al. [16] estimate a 12.99% reporting rate in Brazil as of 20 March 2020.

Our paper is organized as follows. In Section 2, we review the Poisson-logistic model of Winkelmann et al. [3] and the extension to allow for spatial effects by Stoner et al. [7]. We then discuss an extension to the model that allows for sensitivity analysis for a range of false-positive rates. In Section 3, we describe a simulation study to justify the superiority of our extended model. Then, in Section 4, we illustrate the model with data from the first two months of the COVID-19 outbreak in the United States. Finally, we provide concluding thoughts in Section 5. The code used to run the most complicated model is in the Appendix E.

## 2. Methods

### 2.1. Poisson-Logistic Model

Relatively few different approaches have been proposed to account for under-reporting in count data. The most popular method is the Poisson-logistic (Pogit) model proposed by Winkelmann et al. [3]. The model consists of a binomial component for the observed counts, z, conditional on the underlying unobserved true counts, y, which are assumed to follow a Poisson distribution. Adding spatial effects similar to that of Stoner et al. [7], we consider the following initial model that addresses under-reporting,

(1) $$\begin{array}{cc} \; & z_{i}∼\mathrm{Binomial}\left(\pi_{i},y_{i}\right), \end{array}$$

(2) $$\begin{array}{cc} \; & \log\left(\frac{\pi_{i}}{1-\pi_{i}}\right)={W\beta }_{s}, \end{array}$$

(3) $$\begin{array}{cc} \; & y_{i}∼\mathrm{Poisson}\left(E_{i}\lambda_{i}\right), \end{array}$$

(4) $$\begin{array}{cc} \; & \log\left(\lambda \right)={X\gamma }_{s}+\phi +\theta , \end{array}$$

$$\begin{array}{c} \phi ∼N\left(0,\;{\left[\tau \left(D_{w}-W\right)\right]}^{-1}\right), \end{array}$$

where τ is an overall precision parameter, $D_{w}$ is an N×N diagonal matrix whose diagonal element, $d_{ii}$, equals the number of neighbors for region *i*, W is the adjacency matrix with elements $w_{ij}$ equal to 1 if regions *i* and *j* are neighbors (sharing a border) and 0 otherwise. Equation (4) was initially proposed by Besag et al. [8] and referred to as the BYM model. BYM is popular and commonly used for areal data due to its flexibility; however, fitting the BYM model with Markov Chain Monte Carlo (MCMC) methods is difficult due to the non-identifiability of ϕ and θ. Thus, the sampler will often explore many extreme areas of the joint-parameter space. The BYM2 model is a good solution for this difficulty. BYM2 was discussed in detail by Riebler et al. [9] following the penalized complexity framework proposed by Simpson et al. [10]. Like the BYM model, BYM2 includes both spatial and non-spatial random effects. The difference lies in that BYM2 places a single precision parameter, σ, on the combined random effects and uses a mixing parameter, ρ, for the proportion of spatial/non-spatial variation. BYM2 rewrites ϕ + θ in BYM as,

(5) $$\begin{array}{c} \phi +\theta =\sigma \left(\sqrt{\rho }\tilde{\theta }+\sqrt{(1-\rho )/s}\tilde{\phi }\right), \end{array}$$

where σ represents the overall standard deviation, and ρ∈(0,1) controls the proportion of the variation modeled by ICAR, i.e., $\tilde{\phi }$, which is scaled by a rescaling parameter, *s*, such that $var(\tilde{\phi_{i}})\approx 1$. The heterogeneous effect ${\tilde{\theta }}_{i}$ follows Normal(0,n), where *n* is the number of connected subgraphs. Morris et al. [17] proposed to determine the variance as the number of connected subgraphs. In our case, we fixed it at one because the neighborhood graph is fully connected. For ICAR model for disconnected graphs, see Freni-Sterrantino et al. [18]. The quantity, σ, is assumed to follow a Normal(0,1), which requests a condition that $var\left({\tilde{\theta }}_{i}\right)\approx var\left({\tilde{\phi }}_{i}\right)\approx 1$. By using an undirected graph, Morris et al. [17] proposed a novel implementation of BYM2 in the Stan language [11], which substantially reduces the usage of computer memory, and significantly increases the fitting efficiency and speed. Because the scaling factor, *s*, depends on the spatial structure of the specific dataset, it is provided to the Stan model as data. Following Morris et al. [17], we compute *s* using inla.scale.model function in the R package INLA developed by Lindgren et al. [19]. For more details about INLA, please also refer to Rue et al. [20].

We need at least a mildly informative prior on at least one parameter in the logistic regression portion of the model because of the over-parameterization of the Pogit model. One approach to develop this prior is to elicit from an expert or previous data on what the under-reporting would be at the “average” value of all the centered covariates. We denote this reporting probability as $p_{0}$. A beta prior for $p_{0}$ is developed using the prior data and/or expert opinion. A prior for the intercept $\beta_{0}$ is then induced through the logistic relationship. Such a setting is convenient as we can use estimates from other studies or expert opinions on the national or local reporting rates. One can extend this process to obtain a fully informative prior by eliciting beta priors for a range of settings across the covariate space and similarly inducing a joint prior for all the regression parameters. To elicit the beta prior for $p_{0}$, we assume either an expert or reliable prior data is available. In the case of an expert elicitation, a common approach to determine a beta prior is to elicit a central value (such as mean, median, or mode) and a percentile. The expert is asked two questions.

1. What value is most likely for the reporting probability?
2. What value would be considered unusually high?

Setting these two equations equal to, for example, the mode and 99th percentile of a beta distribution CDF yields the parameters of the beta distribution. The R function “elicit_beta” provided in the Appendix E takes the inputs of the mode, quantile, and tail probability and numerically solves for the elicited prior. In this work, we use relatively diffuse normal priors for the other regression parameters. A Normal (0, 1) prior is assigned to logit(ρ), σ and ${\tilde{\theta }}_{i}$, respectively. Relatively non-informative Normal (0, 10) priors are used for $\gamma_{s}$ and $\beta_{s}$.

### 2.2. Model with False Positives

In many real-life applications, including the COVID-19 data of interest here, the counts may contain false positives (FP, i.e., over-reporting) in addition to the under-reporting (false negatives). Here, we outline the additions to the model to account for both false negatives and false positives. We extend the model of Bratcher et al. [21]. For the following, the models for $y_{i}$ and $z_{i}$ are as defined in Equations (1)–(4).

$$\begin{array}{cc} \; & \mathrm{True}\;\mathrm{count}:\;Y{}_{i}∼\mathrm{Poisson}\left(E{}_{i}\lambda {}_{i}\right),\\ \; & \mathrm{TP}\;\mathrm{count}:\;\left(X{}_{i}|Y{}_{i}=y{}_{i}\right)∼\mathrm{Binomial}\left(\pi {}_{i},y{}_{i}\right),\\ \; & \mathrm{FP}\;\mathrm{count}:\;T{}_{i}∼\mathrm{Poisson}\left(E{}_{i}\psi \right), \end{array}$$

where TP are the true positives which have probability $\pi {}_{i}$ of being reported. The number of occurrences that are missed ($Y{}_{i}$−$X{}_{i}$) can be at most $Y{}_{i}$. For the false positives, since the number of tests is large and the probability of a false positive is minimal, we use the Poisson approximation to the binomial for these counts. Again, since the number of tests was large, we can also assume the false positives are independent of the true occurrences.

For the spatial dependency, we assume the same structure as in Section 2.1 for the Poisson rate of the true count, $\lambda {}_{i}$. In the case of the COVID-19 data we model here, we do not have enough data to model the false-positive rates across covariates as we modeled the under-reporting. So, we assume that it is roughly constant across all covariates and areas, and we assign a gamma prior ψ,

$$\begin{array}{c} \psi ∼\mathrm{gamma}(a_{\psi },b_{\psi }). \end{array}$$

An informative prior would typically be required. Using prior information on the number of false positives per test and the testing rate in the population, a gamma prior can be formed. Alternatively, a mode and a percentile for the rate of false positives can be elicited from an expert and converted into the corresponding gamma distribution. Regardless of how the prior is parameterized, the data would provide little information for this particular parameter, so this approach can be viewed as a sensitivity analysis for the overall model, allowing for a range of potential over-reporting along with the under-reporting from the Poisson-logistic model. The function “elicit_gamma” provided in the Appendix E is used to determine a prior for a given mode and percentile, similar to the “elicit_beta” function previously discussed.

## 3. A Simulation Study

We implemented a simulation study to evaluate the performance of the proposed models. To generate the true counts, we considered parameters $\gamma_{s}=\left(5,-1,2\right)$. For the under-reporting rates, we selected parameters $\beta_{s}=c\left(-2,0.5\right)$. Thus, we assumed two covariates for the Poisson regression and one covariate for the under-reporting. All covariates were independently drawn from U(−1,1). Finally, we set the false positive rate ψ=6. One hundred data sets were replicated in this simulation. We use Normal prior N$\left(0,10^{2}\right)$ for $\gamma_{0},\gamma_{1},\gamma_{2}$ and $\beta_{1}$. Instead of directly placing a prior on $\beta_{0}$, we assigned a Beta(2,8) prior on $p_{0}=\exp\left(\beta_{0}\right)/\left[1+\exp\left(\beta_{0}\right)\right]$ to mimic the application to COVID-19 data in Section 4. For the 100 simulated data sets, we considered five different models (explicit forms are listed in Appendix A) for the data. The five models, in order of complexity, are as follows:

$$\begin{array}{cc} \; & M1:\;\mathrm{Naive}\;\mathrm{Poisson}\\ \; & M2:\;\mathrm{Under}-\mathrm{reporting}\;\mathrm{only}\\ \; & M3:\;\mathrm{Spatial}\;\mathrm{only}\\ \; & M4:\;\mathrm{Under}-\mathrm{reporting}\;\mathrm{and}\;\mathrm{spatial}\\ \; & M5:\;\mathrm{Under}-\mathrm{reporting},\;\mathrm{over}-\mathrm{reporting}\;\mathrm{and}\;\mathrm{spatial} \end{array}$$

We provide average posterior bias, mean square error, and coverage probabilities for 95 percent credible intervals for the five models in Table 1. We see that the model with both under-reporting and over-reporting (M5) performs quite well since the average bias and mean square error are small, and the coverage of the 95 percent intervals are close to nominal for all parameters. Models that do not consider spatial dependency (M1 and M2) perform poorly for most parameters, and M2 exhibits a fairly large bias and MSE for some parameters. In addition, the coverages of the 95 percent intervals given by M1 and M2 are all much lower than nominal. It is interesting, but not surprising, that when applying to the simulated data with both under-reporting and over-reporting, M4 performs similar to M5, but not quite as well. Specifically, there is slightly more bias for the parameters of the Poisson regression part of the Pogit model, and the low interval coverage for $\gamma_{2}$ is significant. This finding illustrates that ignoring moderate over-reporting can impact results.

## 4. Application

### 4.1. Data

**Figure 1:**
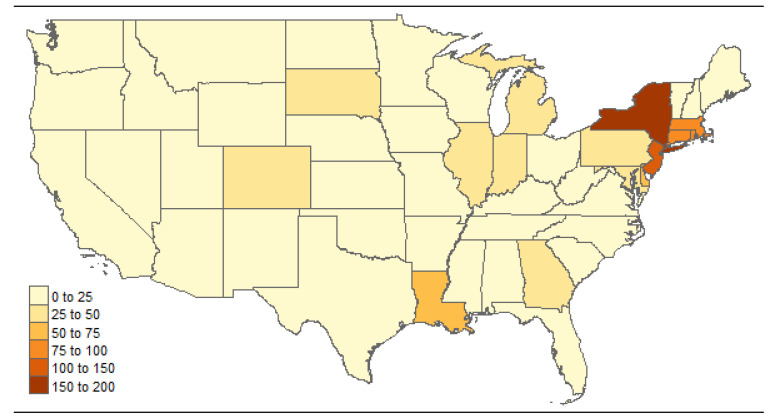
State-level confirmed COVID-19 cases per 10K population as of 30 April 2020.

We collected the state-level accumulated COVID-19 cases of the U.S. from https://covidtracking.com/, accessed on 11 April 2021. Figure 1 shows the state-level map of accumulated confirmed COVID-19 cases per 10,000 people as of 30 April 2020. At this moment in the pandemic, 1,071,003 confirmed cases have been reported in the 48 contiguous U.S. states and Washington DC. We observed that states in the northeast have a considerably larger number of cases per 10,000 people. In particular, the highest incidence of COVID-19 was reported in New York state (304,372 cases in total, 154 per 10,000 population). According to the global Moran’s I statistic (Moran’s I=0.21, p<0.0001), the state-level COVID-19 counts display highly positive auto-correlation. We also collected state-by-state risk factors from https://www.americashealthrankings.org, accessed on on 11 April 2021. It is believed that insufficient COVID-19 testing capability at the early stage of the pandemic was the key factor leading to under-reporting. Historical testing data by states were also available from https://covidtracking.com, accessed on 11 April 2021. Table 2 presents the description and summary statistics of variables used for the application. In Table 2, the variable “Pop” divided by 1,000,000 is used as the offset *E*, “Testing” is the single covariate used for the reporting procedure, and all the other variables are potential covariates for the Poisson regression. We are interested in actual counts of COVID-19 cases, not deaths. However, early in the pandemic, often only more severe cases were reported, which is why we included several covariates which were considered to be related to underlying medical conditions such as “Smoking”, “Obesity”, and “Air pollution” (strongly related to asthma). Furthermore, “Alcoholism”, “Inactivity”, and “Drug deaths” are included as we believe these behavioral factors may have a potential impact on the willingness or awareness to get tested when symptoms are mild. Furthermore, free testing was very limited in the first two months; thus, people without insurance would be less likely to request the COVID-19 test as the fees may have been unaffordable, so “uninsured” is included.

### 4.2. Priors

The prior distributions for the parameters can be developed through expert opinion or induced from other studies. As mentioned previously, we require at least one mildly informative prior so that the posterior distributions converge appropriately. The conditional means prior approach of Bedrick et al. [22] can be used to obtain a fully informative prior for all regression parameters. Here, we use it to get an informative prior for the intercept of the logistic regression. We first assume diffuse normal priors for all the regression parameters except $\beta_{0}$,

(6) $$\begin{array}{cc} \beta_{s} & ∼N(0,10^{2}),\;s=1,\cdots ,k-1 \end{array}$$

(7) $$\begin{array}{cc} \gamma_{s} & ∼N(0,10^{2}),\;s=0,1,\cdots ,j-1, \end{array}$$

where, *k* and *j* are numbers of covariates used for the logistic and Poisson components, respectively. To determine an informative prior for $\beta_{0}$, we refer to the available studies on COVID-19 reporting rates. The references in Section 1 indicate that the actual reporting rate in the early stage of the pandemic was meager, so we assume that the mode of the probability at the average value of the covariates, $\pi_{0}$, to be around 0.1 and that $Pr(\pi_{0}>0.3)$ is small ( i.e., $1-Pr(\pi_{0}<=0.3)=0.0001$) which results in a beta(7,55) prior. Because all covariates are centered, $\beta_{0}$ is interpreted as the log-odds of the reporting rate at the average level of the covariates. In this case if we assume $logit\left(p_{0}\right)=\beta_{0}$, the beta(7,55) prior on $p_{0}$ induces a prior on $\beta_{0}$. Note that a Jacobian adjustment is necessary for the log-likelihood statement in Stan. This approach of prior elicitation could be extended to other regression parameters by eliciting priors on the probabilities for a range of covariate values spread across the covariate space. Then, this idea is used to induce a prior on all the coefficients similar to the method we used just for the intercept. We also considered a beta(5,78) prior, which corresponds to an expected reporting probability of only 5% but could be as high as 20%. For the model with false positives, we require an informative prior for ψ. We are modeling the false positive rate on the same scale as observed counts. We are looking at false positives per population, not per a certain number of tests. This approach allows the model to be generalizable to other situations where counts are not the result of a certain number of diagnostic tests. As of April 2020, testing was still relatively low, so we consider two priors for the false positive rate, a gamma (5, 1) which would correspond to an expected five false positives per 100,000 people, and gamma (30, 1), which corresponds to an expected 30 false positives per 100,000 people. The modeling process is implemented in R [23] and Stan. The code is provided in the Appendix E.

### 4.3. Results

Our simulation in Section 3, along with the simulation results in Stoner et al. [7], confirm the usefulness of the hierarchical model. Specifically, ignoring the under-reporting and spatial aspects of the data result in biased estimates and underestimation of posterior variability. The results of the analysis of the COVID-19 data are summarized in Table 3. As expected, M1 and M2 exhibit considerably less posterior variability as they ignore the significant spatial dependency. The results of M3 are likely to be an improvement, but from the simulation studies, the posterior means are likely biased since the under-reporting is not considered. M4 and M5 have very similar estimates of most regression parameters and spatial random effects (see Appendix D). Both show “Testing” is a strong factor that is positively related to the COVID-19 case reporting rate. The deviance information criterion (DIC) for M1–M5 are 152,001, 131,129, 633.1, 631.1, and 631.3, respectively. Thus, M3, M4, and M5 provide very similar fits to the data, but there seems to be a preference for M4, which accounts for under-reporting but ignores any potential over-reporting. This result is likely reasonable as the degree of under-reporting is so large it almost certainly washes out the impact of whatever over-reporting happened to be present in the early days of the pandemic. The best fit model, M4, has three significant variables in the Poisson component of the model for the COVID-19 counts. These variables are “Alcoholism”, “Smoking”, and “Inactivity”. The effects of “Alcoholism” and “Inactivity” are positive, while the effect of “Smoking” is negative. These results indicate that high alcoholism and inactivity percentages may increase the state-level incidence of COVID-19 while smoking is protective. The latter is surprising as smoking is a risk factor for respiratory diseases. This scenario is also possible as an example of the ecological fallacy since the data is collected at the state level. Our model can estimate the central tendency and credible set of the reporting rate for each state (see Appendix C).

The simulation and application are both implemented with the R and Stan languages. It is worth mentioning that we obtain relatively stable (convergent) results with a burn-in of only 2000 iterations and 4000 iterations in this application which is much faster and more efficient than Stoner et al. [7], in which 400,000 burn-ins and 800,000 iterations were required for convergent chains used for inference (they use the Nimble package in R).

To check prior sensitivity, we reran model 5 with all combinations of the priors used for the reporting probability and false-positive rate. That is the beta (7, 55) and beta (5, 78) that induce an informative prior for the logistic model and the gamma (5, 1) and gamma (30, 1) for the false positive rate. The results were remarkably robust concerning these choices of prior. No posterior loses or gains statistical significance due to changes in the priors, and the posterior means change very little across the prior distribution combinations. For instance, the posterior means for the alcoholism coefficient ranged from 0.201 to 0.213 across the four combinations while the posterior for inactivity ranged from 0.413 to 0.427. The summaries for all the coefficients are provided in Appendix B.

### 4.4. Model Checking

**Figure 2:**
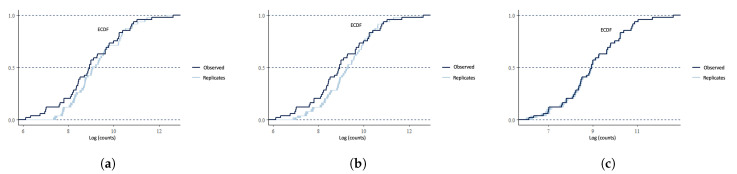
Comparison between the empirical distribution of the logarithmic observed COVID-19 counts and the distributions of simulated/replicated samples from the posterior predictive distribution based on M1, M3, and M4. (**a**) M1: naive Poisson model; (**b**) M2: under-reporting only; and (**c**) M4: under-reporting and spatial.

We visualize the empirical cumulative distribution functions (ECDF) of observed counts and posterior predictive samples for M1, M2, and M4 in Figure 2, where the ECDFs are plotted on the log scale for better visualization. The dark blue solid line in each subplot is the ECDF of observed responses, while the lighter blue lines are the ECDFs of 200 randomly selected posterior predictive samples given by each model. We see an apparent deviation between the observed ECDF and the predictive ECDFs in (a) and (b). In contrast, (c) shows a substantial overlap of the observed ECDF and the predictive ECDFs. Like the DIC, the check based on the posterior predictive favors M4 that considers both under-reporting and spatial dependency.

## 5. Conclusions

Misclassification in the form of under-reporting and over-reporting can bias estimates and potentially lead to wrong decisions. When the decisions are for issues related to a worldwide pandemic, it is of particular importance that all tools available are applied to bring the best information available to policymakers. This paper has used and extended a recently proposed Bayesian model that accounts for under-reporting and spatial effects. Our extensions include allowing for potential over-reporting, a suggested way to include experts’ prior information, and coding the model in an alternative software that appears to result in much faster results.

For the COVID-19 data, we found that accounting for the under-reporting yielded relatively similar inferences, with the main difference in the effect sizes. That is, no parameter estimates switched direction, only magnitude. One can easily extend the model by adding a time component if repeated counts are available, allowing for estimation and prediction of the trajectories of the count variable in each area. On the other hand, we could expect more accurate inference if the model is applied to higher spatial resolution data, such as county-level COVID-19 cases, which is not complex as the computation is speedy due to the clever implementation of the BYM2 in Stan language.

This study is confined to modeling spatial variation in adjusting for misreporting. Infectious disease data sets are often recorded at high temporal resolutions and used to study characteristics of seasonal epidemics and future pandemics. Notably, it is crucial to timely and accurately forecast the characteristics of emerging infectious diseases in public health. Thus, our future study could be extending the proposed models to Spatio-temporal misreporting models that account for spatial and temporal variations simultaneously.

Another limitation is we did not include spatial effects on the reporting mechanism itself. This direction is another essential extension that will require significant work as additional identifiability constraints will need to be resolved.

## Figures and Tables

**Table 1:** Summaries of simulation for five models, $\beta_{0}=-2$, ψ=6.

		Average Bias					MSE					Coverage				
Parameter	Truth	M1	M2	M3	M4	M5	M1	M2	M3	M4	M5	M1	M2	M3	M4	M5
$\beta_{0}$	−2	(N/A)	0.672	(N/A)	0.252	**0.24**	(N/A)	9.697	(N/A)	0.082	**0.076**	(N/A)	0.33	(N/A)	**1**	**1**
$\beta_{1}$	0.5	(N/A)	0.958	(N/A)	**0.046**	0.076	(N/A)	9.844	(N/A)	**0.079**	0.087	(N/A)	0.2	(N/A)	**1**	**1**
$\gamma_{0}$	5	−0.121	0.456	−0.213	0.102	**0.004**	0.019	0.558	0.047	0.012	**0.002**	0.06	0.21	0	**1**	**1**
$\gamma_{1}$	−1	−0.018	−0.024	0.065	0.061	**−0.021**	0.025	0.027	0.01	0.009	**0.008**	0.15	0.1	0.89	0.91	**0.96**
$\gamma_{2}$	2	−0.073	−0.075	−0.161	−0.162	**−0.016**	0.036	0.035	0.031	0.031	**0.007**	0.11	0.11	0.48	0.45	**0.99**
ψ	6	(N/A)	(N/A)	(N/A)	(N/A)	−0.497	(N/A)	(N/A)	(N/A)	(N/A)	1.635	(N/A)	(N/A)	(N/A)	(N/A)	1

**Table 2:** Description and summary statistics of variables used for state-level COVID-19 cases.

Variable	Description	Max	Min	Mean	Median	sd
AirPollution	The average exposure of the general public to particulate matter of 2.5 microns or less measured in micrograms per cubic meter (3-year estimate)	12.80	4.40	7.48	7.40	1.45
Uninsured (%)	Percentage of population not covered by private or public health insurance	17.50	2.80	8.09	8.10	2.99
Inactive (%)	Percentage of adults who reported doing no physical activity or exercise other than their regular job in the past 30 days	32.40	16.40	24.17	23.80	3.84
Obesity (%)	Percentage of adults with a body mass index of 30.0 or higher based on reported height and weight	39.50	22.90	31.46	30.90	3.86
Smoking (%)	Percentage of adults who reported smoking at least 100 cigarettes in their lifetime and currently smoke daily or some days	25.20	9.00	16.61	16.10	3.32
Alcoholism (%)	Percentage of adults who reported binge drinking (four or more (women) or five or more (men) drinks on one occasion in the past 30 days) or heavy drinking (eight or more (women) or 15 or more (men) drinks per week)	26.30	11.30	18.17	18.20	3.10
Drug deaths	Number of deaths due to drug injury (unintentional, suicide, homicide or undetermined) per 100,000 population	48.30	7.20	20.78	19.90	8.87
MDI	An index of seventeen socioeconomic indicators from the American Community Survey 5-year sample at the block group level	21.45	8.23	13.81	13.48	3.43
Popdensity	Population per square mile	11,011.00	6.00	424.33	106.00	1566.86
Pop	Population	39,144,818	586,107	6,515,301	4,670,724	7,268,509
Testing	The total number of testing per 1000 people as of the cut-off date	62.81	10.86	22.09	17.95	11.03

**Table 3:** Posterior mean (90% CI) for coefficients of COVID-19 data obtained from five different models. Significant results are bolded.

Variable	M1	M2	M3
Intercept_1_(γ_0_)	7.79(7.788,7.793)	8.242(8.235,8.249)	7.614(7.516,7.71)
Uninsured ($\gamma_{1}$)	−0.448(−0.451,−0.446)	−0.352(−0.355,−0.349)	−0.188(−0.379,0.005)
Obesity ($\gamma_{2}$)	−0.377(−0.382,−0.373)	−0.323(−0.328,−0.318)	−0.171(−0.418,0.07)
Alcoholism ($\gamma_{3}$)	0.001(−0.002,0.004)	0.005(0.002,0.008)	0.18(−0.016,0.364)
AirPollution ($\gamma_{4}$)	−0.422(−0.423,−0.42)	−0.301(−0.304,−0.299)	−0.14(−0.333,0.057)
Drug deaths ($\gamma_{5}$)	−0.018(−0.021,−0.015)	−0.055(−0.058,−0.052)	0.009(−0.208,0.221)
MDI ($\gamma_{6}$)	0.329(0.326,0.331)	0.221(0.218,0.223)	0.134(−0.072,0.349)
Smoking ($\gamma_{7}$)	−0.449(−0.454,−0.445)	−0.291(−0.296,−0.286)	−0.583(−0.859,−0.317)
Inactivity ($\gamma_{8}$)	0.637(0.634,0.64)	0.51(0.507,0.514)	0.552(0.258,0.852)
Popdensity ($\gamma_{9}$)	0.045(0.041,0.049)	0.036(0.032,0.039)	0.029(−0.176,0.226)
Intercept${}_{2}$ ($\beta_{0}$)	(N/A)	1.165(1.139,1.192)	(N/A)
Testing ($\beta_{1}$)	(N/A)	1.624(1.603,1.646)	(N/A)
DIC	152,001	131,129	633.1
	M4	M5	
Intercept${}_{1}$($\gamma_{0}$)	9.658(9.166,10.224)	9.659(9.152,10.218)	
Uninsured ($\gamma_{1}$)	−0.098(−0.271,0.069)	−0.096(−0.271,0.078)	
Obesity ($\gamma_{2}$)	−0.198(−0.398,0.01)	−0.203(−0.407,0.004)	
Alcoholism ($\gamma_{3}$)	0.205(0.026,0.376)	0.209(0.026,0.394)	
AirPollution ($\gamma_{4}$)	0.02 (−0.181,0.216)	0.017 (−0.178,0.228)	
Drug deaths ($\gamma_{5}$)	−0.076(−0.272,0.116)	−0.068(−0.268,0.117)	
MDI ($\gamma_{6}$)	0.039 (−0.15,0.23)	0.043 (−0.15,0.235)	
Smoking ($\gamma_{7}$)	−0.357(−0.616,−0.104)	−0.367(−0.624,−0.096)	
Inactivity ($\gamma_{8}$)	0.407(0.146,0.671)	0.407(0.148,0.667)	
Popdensity ($\gamma_{9}$)	−0.028(−0.219,0.165)	−0.033(−0.217,0.166)	
Intercept${}_{2}$ ($\beta_{0}$)	−1.882(−2.493,−1.298)	−1.887(−2.508,−1.285)	
Testing($\beta_{1}$)	0.414(0.198,0.658)	0.418(0.184,0.673)	
DIC	631.1	631.3	

**Figure A1:**
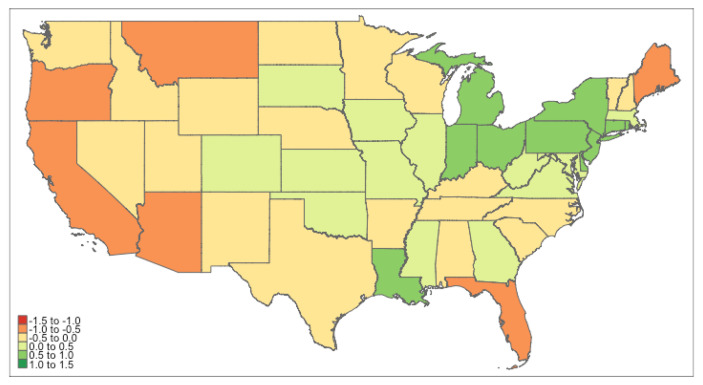
Map of posterior median spatial random effects obtained by M3.

**Figure A2:**
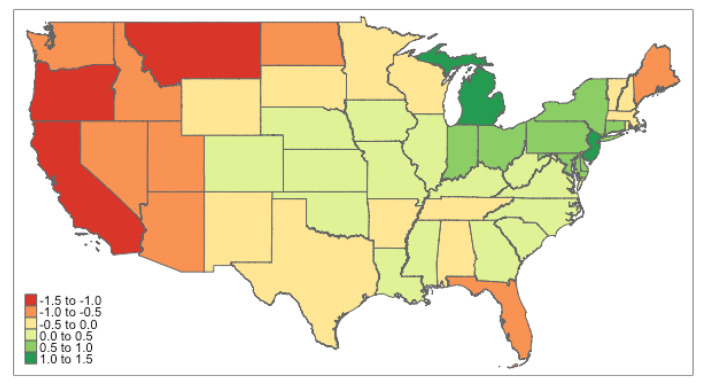
Map of posterior median spatial random effects obtained by M4.

**Figure A3:**
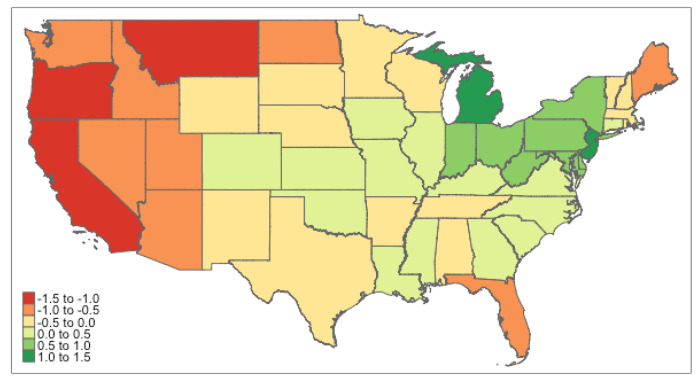
Map of posterior median spatial random effects obtained by M5.

## Data Availability

The data is available at https://covidtracking.com/ (accessed on 11 April 2021) and https://www.americashealthrankings.org (accessed on 11 April 2021).

## Appendix A. Explicit Forms for M1 to M5

$$\begin{array}{cc} M1:\; & z_{i}∼\mathrm{Poisson}\left(E_{i}\lambda_{i}\right)\\ \; & \log\left(\lambda \right)={X\gamma }_{s}+\theta \\ M2:\; & z_{i}∼\mathrm{binomial}\left(\pi_{i},y_{i}\right)\\ \; & \log\left(\frac{\pi_{i}}{1-\pi_{i}}\right)={W\beta }_{s}\\ \; & y_{i}∼\mathrm{Poisson}\left(E_{i}\lambda_{i}\right)\\ \; & \log\left(\lambda \right)={X\gamma }_{s}+\theta \\ M3:\; & z_{i}∼\mathrm{Poisson}\left(E_{i}\lambda_{i}\right)\\ \; & \log\left(\lambda \right)={X\gamma }_{s}+\phi +\theta \\ M4:\; & z_{i}∼\mathrm{binomial}\left(\pi_{i},y_{i}\right)\\ \; & \log\left(\frac{\pi_{i}}{1-\pi_{i}}\right)={W\beta }_{s}\\ \; & y_{i}∼\mathrm{Poisson}\left(E_{i}\lambda_{i}\right)\\ \; & \log\left(\lambda \right)={X\gamma }_{s}+\phi +\theta \\ M5:\; & z_{i}=x_{i}+t_{i}∼\mathrm{Poisson}\left(E_{i}\left(\lambda_{i}\pi_{i}+\psi \right)\right)\\ \; & y_{i}∼\mathrm{Poisson}\left(E_{i}\lambda_{i}\right)\\ \; & x_{i}∼\mathrm{binomial}\left(\pi_{i},y_{i}\right)\\ \; & t_{i}∼\mathrm{Poisson}\left(E_{i}\psi \right)\\ \; & \log\left(\frac{\pi_{i}}{1-\pi_{i}}\right)={W\beta }_{s}\\ \; & \log\left(\lambda \right)={X\gamma }_{s}+\phi +\theta \end{array}$$

## Appendix B. Sensitivity Analysis for M5

**Table A1:** Sensitivity analysis for M5 with different priors on intercept${}_{2}$ $\beta_{0}$ and false-positive rate ψ.

Priors	Beta (7, 55), Gamma (5, 1)			Beta (5, 78), Gamma (5, 1)			Beta (5, 78), Gamma (30, 1)			Beta (7, 55), Gamma (30, 1)		
	**M ^1^**	**L**	**U**	**M**	**L**	**U**	**M**	**L**	**U**	**M**	**L**	**U**
Intercept${}_{1}$	9.653	9.133	10.253	10.253	9.643	10.956	10.213	9.653	10.863	9.635	9.145	10.209
Uninsured	−0.103	−0.278	0.078	−0.101	−0.287	0.072	−0.102	−0.286	0.072	−0.097	−0.289	0.095
Obesity	−0.199	−0.415	0.02	−0.196	−0.423	0.017	−0.196	−0.403	0.021	−0.205	−0.426	0.013
Alcoholism	0.204	0.023	0.387	0.201	0.022	0.377	0.209	0.029	0.389	0.213	0.037	0.39
AirPollution	0.012	−0.182	0.208	0.005	−0.191	0.217	−0.002	−0.205	0.191	0.018	−0.182	0.218
Drug_death	−0.07	−0.263	0.119	−0.07	−0.265	0.117	−0.065	−0.275	0.138	−0.071	−0.27	0.128
MDI	0.038	−0.147	0.242	0.049	−0.147	0.236	0.048	−0.154	0.251	0.038	-0.158	0.234
Smoking	−0.369	−0.629	−0.098	−0.375	−0.639	−0.092	−0.392	−0.653	−0.129	−0.37	−0.63	−0.107
Inactivity	0.417	0.149	0.666	0.415	0.149	0.683	0.427	0.152	0.7	0.413	0.129	0.694
Popdensity	−0.025	−0.216	0.17	−0.023	−0.216	0.169	−0.022	−0.227	0.182	−0.034	−0.213	0.146
Intercept${}_{2}$	−1.88	−2.572	−1.254	−2.557	−3.301	−1.891	−2.53	−3.224	−1.898	−1.877	−2.525	−1.294
Testing	0.415	0.187	0.682	0.358	0.176	0.572	0.362	0.162	0.579	0.426	0.202	0.688

^1^ M, L, and U are shorts for the posterior median, 90% lower and upper bound, respectively.

## Appendix E. Codes

### Appendix E.1. Stan Code for Model 5

covid19_FP_under_BYM2 <–

// *function of ICAR following Morris~et~al.*

functions {

       real icar_normal_lpdf ( vector phi, **int** N,  **int** [ ]  node 1,

        **int** [ ]  node 2 ) {

       **return**  –0.5  *  dot_self ( phi [node1] – phi [node2] )+

               normal_lpdf (sum( phi ) | 0 ,0.001  * N);}

   }

   data  {

     **int** <lower=0> N; // *number of regions*

     **int** <lower=0> N_edges ; // *number of edges*

     **int** <lower=1, upper=N> node1[N_edges]; // *number of node 1*

     **int** <lower=1, upper=N> node2[N_edges]; // *number of node 2*

     **int** <lower=0> z[N] ; // *observed counts*

     vector <lower=0>[N] E; // *exposures*

     **int** <lower=1> K; // *number of covariates for Poisson regression*

     matrix [N,K] X; // *design matrix for Poisson regression*

     **int** <lower=1> J; // *number of covariates for Logistic regression*

     matrix [N, J] W; // *design matrix for Logistic regression*

    // *scaling the variance of the spatial effects*

    real <lower=0>  scaling_factor ;

    // *hyper-parameters for false positive parameter Psi*

    real <lower=0>  prior_a ;

    real <lower=0>  prior_b ;

}

transformed data {

     vector [N] logE=log ( E );

}

parameters  {

// *coefficients for Poisson regression*

  real gamma0;

  vector [ K ] gammas;

// *coefficients for Logistic regession*

  real beta0;

  vector [ J ] betas;

  real logit_rho ; // *proportion of spatial variance*

  vector [N] phi ; // *spatial random effects*

  vector [N] theta ; // *heterogeneous effects*

  real <lower=0> sigma ; // *overall standard deviation*

  real <lower=0> Psi ; // *overall false positive rate*

}

transformed parameters{

  // *Average reporting rate when W are centralized*

   real <lower=0, upper=1>  p0;

   vector <lower=0, upper=1>[N] p;  // *reporting rates*

   vector <lower=0> [N] lambda ;  //  *true Poisson rates*

  // *Poisson rates with under- and over-reporting*

   vector <lower=0>[N]  mu;

   real <lower=0, upper=1>  rho = inv_logit ( logit_rho );

   vector [N] convolved_re;

   convolved_re = sqrt ( 1–rho ) * theta +sqrt ( rho / scaling_factor ) * phi;

   lambda = exp ( gamma0 + X * gammas + convolved_re * sigma );

   p0 = inv_logit ( beta0 );

   p = inv_logit ( beta0 + W*betas );

   mu = lambda  .* p  +  Psi ;

}

model  {

//  *likelihood*

  z ~ poisson ( mu.*  E );

  target += beta0–2*log ( 1 + exp ( beta0 ) ) ;// *Jacobian adjustment*

// *priors*

  p0  ~  beta ( 7 , 55 ) ;

  gammas ~ normal ( 0 , 10 ) ;

  gamma0 ~ normal ( 0 , 10 ) ;

  betas ~ normal ( 0 , 10 );

  logit_rho ~ normal ( 0 , 1 );

  sigma ~ normal ( 0 , 1 ) ;

  theta ~ normal ( 0 , 1 ) ;

  phi ~ icar_normal (N,  nod1 ,  node2 ) ;

  Psi ~ gamma  ( prior_a , prior_b ) ;

}

generated  quantities{

  vector [N]  log_lik ;  //*loglikelihood*

  vector [N]  eta = gamma0 + x*gammas + convolved_re * sigma;

  vector [N]  lambda_rep = exp ( eta ) ;

  vector [N]  p_rep  =  inv_logit ( beta0 + w * betas ) ;

  vector [N] mu_rep;  //*posterior samples of mu*

  **int** z_rep [N] ;

  **for** (n  in  1:N){

     mu_rep [n] = lambda_rep [n] * p_rep [n] + Psi;

     //  *compute the po**int**-wise log likelihood*

     log_lik [n] = poisson_lpmf ( z [n]  |  mu [n] );

  }

  **if** ( max ( eta ) <20 ) {  //  *avoid overflow in poisson_log_rng*

    **for** ( n in 1:N)  {

     z_rep [n]  =  –1;

    }

  } **else** {

    **for** ( n in 1:N)  {

      z_rep [n] = poisson_rng ( mu_rep [n] .* E );

      }

  }

}

### Appendix E.2. R Code for Beta_elicit Function

# mode is most likely elicited value

# c is the percentile

# alpha is the tail probability ( i.e., percentage in the tail

# cutoff  by  c )

beta_elicit <– function ( mode,  c,  alpha ) {

 b <– 1 # initial value

 a <– ( 1 + mode * ( b–2 )) / ( 1–mode )

 f <– function ( b ) {

  a <– ( 1 + mode * ( b–2 ) ) / ( 1–mode )

  qbeta  ( 1–alpha, a, b )–c

 }

 x <– uniroot ( f ,  lower=1,  upper=100 )

 b <– x$root

 a <– ( 1 + mode * ( b–2 ) ) / ( 1 –mode )

 list ( a,  b )

}

# If expert thinks most likely value is 0.2,

# 99% sure that value is below .5

# function would be beta_elicit ( .2, .5, .01 )

beta_elicit ( .2 , .5 , .01 )

beta_elicit ( .9 , .7 , .95 , 2 )

### Appendix E.3. R Code for Gamma_elicit Function

# mode is most likely elicited value

# c is the percentile

# alpha is the tail probability ( i.e.,  percentage in the tail

# cutoff  by  c )

gamma_elicit <- function ( mode,  c,  alpha ) {

 b <– 1 # initial value

 a <– 1 + b * mode

 f <– function ( b ) {

    qgamma ( 1 – alpha,  a,  b )–c

 }

 x <– uniroot ( f ,  lower = 0 ,  upper = 25 )

 b <– x$root

 a <– 1 + b * mode

 list ( a,  b )

}

gamma_elicit ( 10,  30,  .05 )
